# Genotyping of French *Bacillus anthracis* Strains Based on 31-Loci Multi Locus VNTR Analysis: Epidemiology, Marker Evaluation, and Update of the Internet Genotype Database

**DOI:** 10.1371/journal.pone.0095131

**Published:** 2014-06-05

**Authors:** Simon Thierry, Christophe Tourterel, Philippe Le Flèche, Sylviane Derzelle, Neira Dekhil, Christiane Mendy, Cécile Colaneri, Gilles Vergnaud, Nora Madani

**Affiliations:** 1 University Paris-Est, Anses, Animal Health Laboratory, Bacterial Zoonosis Unit, Maisons-Alfort, France; 2 Univ Paris-Sud, Institut de Génétique et Microbiologie, UMR 8621, Orsay, France; 3 CNRS, Orsay, France; 4 Division of Analytical Microbiology, DGA CBRN Defence, Vert le Petit, France; 5 DGA/MRIS- Mission pour la Recherche et l'Innovation Scientifique, Bagneux, France; Charité-University Medicine Berlin, Germany

## Abstract

**Background:**

*Bacillus anthracis* is known to have low genetic variability. In spite of this lack of diversity, multiple-locus variable-number tandem repeat (VNTR) analysis (MLVA) and single nucleotide polymorphisms (SNPs) including the canonical SNPs assay (canSNP) have proved to be highly effective to differentiate strains. Five different MLVA schemes based on a collection of 31 VNTR loci (MLVA8, MLVA15, MLVA20, MLVA25 and MLVA31) with increased resolving power have been described.

**Results:**

MLVA31 was applied to characterize the French National Reference Laboratory collection. The total collection of 130 strains is resolved in 35 genotypes. The 119 veterinary and environmental strains collection in France were resolved into 26 genotypes belonging to three canSNP lineages and four MLVA clonal complexes (CCs) with particular geographical clustering. A subset of seven loci (MLVA7) is proposed to constitute a first line assay. The loci are compatible with moderate resolution equipment such as agarose gel electrophoresis and show a good congruence value with MLVA31. The associated MLVA and SNP data was imported together with published genotyping data by taking advantage of major enhancements to the MLVAbank software and web site.

**Conclusions:**

The present report provides a wide coverage of the genetic diversity of naturally occurring *B. anthracis* strains in France as can be revealed by MLVA. The data obtained suggests that once such coverage is achieved, it becomes possible to devise optimized first-line MLVA assays comprising a sufficiently low number of loci to be typed either in one multiplex PCR or on agarose gels. Such a selection of seven loci is proposed here, and future similar investigations in additional countries will indicate to which extend the same selection can be used worldwide as a common minimum set. It is hoped that this approach will contribute to an efficient and low-cost routine surveillance of important pathogens for biosecurity such as *B. anthracis*.

## Introduction

*Bacillus anthracis* is a spore forming Gram positive bacterium that causes anthrax, a zoonosis with a worldwide distribution. Anthrax is an acute infectious disease that may impact livestock, wildlife and humans. The zoonosis is still endemic in many countries. Animals, especially ruminants, are infected by ingestion of soilborne spores while grazing [1]. In France, animal cases are regularly reported [2]. A few sporadic cases are recorded each year in areas where outbreaks have been reported in the past, and larger outbreaks occur every few years. Anthrax is a professional disease and humans are infected through exposure to animals or contaminated animal products when such material is incidentally ingested, inhaled or comes into contact with an open wound [3], [4]. *B. anthracis* is also considered as a major potential biowarfare agent.

The currently known *B. anthracis* population displays a low genetic variability and highly clonal evolution [5], [6], [7] indicative of a most recent common ancestor (MRCA) living less than a few tens of thousands years ago [8]. In the last decade, and owing to the development of whole genome sequencing technologies, an exhaustive exploration of genetic polymorphisms was achieved. Two classes of genetic markers are mostly used, variable number of tandem repeats (VNTRs) and single nucleotide polymorphisms (SNPs). These polymorphisms are assayed either by polymerase chain reaction (PCR) targeted analysis of a list of VNTR or SNP loci or by whole genome sequencing [6], [9], [10], [11], [12], [13], [14]. Each of the two classes of genetic variations exhibit very different resolving potentialities and phylogenetic accuracy, related to their own specific mutation rate and number of possible allelic states: from the highly stable but with individually low resolution SNP to the more variable and homoplasic but with fine-scale resolution tandem repeat loci [15].

Thirty-one VNTR loci have been described in the *B. anthracis* genome (not including VNTRs with a one base-pair repeat unit, or Single Nucleotide Repeats (SNRs)) [14]. Several selections among these VNTR loci have been proposed to characterize *B. anthracis* [6], [8], [10], [11], [15], [16]. In year 2000, the first designed MLVA system (MLVA8) targeted six chromosomal (*vrrA*, *vrrB1*, *vrrB2*, *vrrC1*, *vrrC2*, *CG3*) and two plasmid loci (*pXO1*, *pXO2*). It was able to subtype a collection of over 400 *B. anthracis* strains into 89 genotypes defining two major clonal lineages (A and B) [6]. Since then, this VNTR panel has been applied in numerous studies to examine the diversity of *B. anthracis* throughout the world [17], [18], [19], [20], [21], [22], [23], [24], [25]. It is a very robust and convenient assay, in spite of its limited discriminatory power for epidemiologic analysis. However it includes two loci located on the plasmids with two drawbacks. Firstly the plasmids are sometimes absent in environmental strains as well as some collection strains [26], [27] and secondly both loci have short (2 and 3 bp) repeat units, and consequently require high precision DNA fragment sizing equipment.

In a search for higher discriminatory power, additional VNTR loci have subsequently been identified. Le Flèche et al. [10] developed the Microbial Tandem Repeats database (http://minisatellites.u-psud.fr) to facilitate the identification and selection of tandem repeats and used it to propose a MLVA20 scheme for *B. anthracis* including the MLVA8 loci minus the two plasmid markers and 14 newly described polymorphic markers. The MLVA15 scheme designed by Keim and col. [8], [15] is based upon the MLVA8 selection and seven additional loci (vntr12, 16, 17, 19, 23, 32 (alias bams01 from [10]) and vntr35). Lista et al. [11] developed a 25 VNTR markers scheme (MLVA25), including MLVA8, plus 13 of the 14 additional loci proposed by Le Flèche et al. [10] (bams01, 03, 05, 13, 15, 21, 22, 23, 24, 25, 28, 30 and 31; not included: bams07), and 4 new ones (bams34, 44, 51 and 53). The full set of 31 VNTR markers (MLVA31) present in the MLVA8, MLVA15 or MLVA25 panels, has been recently applied in concert with the typing of the set of SNPs called canSNPs [8] to evaluate the genotypic diversity found in Namibia [16]. MLVA provides each genotype in the form of a numeric code that can be easily stored in a database for comparison, as illustrated by the publicly accessible online database (MLVAbank at http://mlva.u-psud.fr/) [28], [29] compiling the numerous genotypes identified in different studies [6], [11], [16], [17], [19], [20], [30], [31], [32].

Taking advantage of the knowledge and data accumulated in the past thirteen years, the main aims of the present study are (1) to provide an update of the genetic diversity of *B. anthracis* strains naturally present in France, using the most recently developed MLVA31 assay, (2) to propose a selection of VNTR loci which could represent a reasonable first-line assay for *B. anthracis* MLVA genotyping (3) to present the new *B. anthracis* genotyping database made by taking advantage of major developments to the underlying MLVAbank software. The first-line assay should contain a minimum number of loci, compatible with typing using a large variety of DNA sizing techniques including basic agarose gel electrophoresis. In the present study, we have used the MLVA31 scheme in combination with canSNP analysis [7], [8] on the complete ANSES collection of strains collected from different regions of France. The discriminatory power of the MLVA31 scheme and the contribution of individual VNTR markers were evaluated to optimize the number of markers required to accurately resolve the genetic diversity found across French strains. Congruence and linkage disequilibrium analysis were conducted for different panels of VNTR marker [33] and the different MLVA schemes were compared. Because databases are a key issue in terms of genotyping, we have significantly improved the software behind the http://mlva.u-psud.fr prototype by introducing three major functionalities: firstly, the new version is able to merge a number of independently curated databases so that they can be queried and browsed as a single entity, secondly, the database can host any kind of numeric genotyping data, such as canSNPs, and thirdly, a tool has been included to automatically deduce a MLVA or SNP genotype from genome sequence data.

## Materials and Methods

### Ethics statement

The strains used in this study were isolated by the French National Reference Laboratory (NRL) for animal anthrax. The NRL is a public laboratory, mandated by the Ministry of Agriculture to confirm diagnosis of all animal anthrax cases in France. During an outbreak, samples are taken by the veterinary services of the Ministry of Agriculture. Specific permission for soil sampling was not required. No human strains were included in this study.

### Bacterial strains

A total of 130 *B. anthracis* strains, including eleven strains from external collections (ten strains acquired from the “Collection de l'Institut Pasteur” (CIP, Paris, France) (17JB, CIP 53.169, CIP 66.17, CIP 74.12, CIP 77.2, CIP 81.89, CIP A204, CIP A205, CIP A206, CIP A211), one strain of uncertain origin from the “Institut d'Élevage et de Médecine Vétérinaire des pays Tropicaux” (IEMVT 89–1620)) and 119 strains collected in France between 1982 and 2010, were included in this study. One hundred and six of the 119 strains are from animal origin: bovine (n = 101), ovine (n = 2), equine (n = 2) and caprine (n = 1). The last thirteen were collected from the environment.

### VNTR typing

The previously described MLVA31 scheme [16] was used. This collection of 31 loci represents all VNTR loci in the MLVA8, MLVA15 and MLVA25 schemes [6], [11], [15] (Table 1). The PCR reactions for the MLVA25 loci were performed in four multiplex reactions A–D using the PTC200 thermocycler (Bio-Rad, Marnes-la-Coquette, France)) as described by Lista et al. [11]. Multiplex A amplifies vrrB2, vrrC1, CG3, bams01 (alias vntr32), bams03, bams05, bams15, bams44. Multiplex B amplifies vrrB1, vrrC2, bams13, bams28, bams31, bams53. Multiplex C amplifies vrrA, bams21, bams24, bams25, bams34. Multiplex D amplifies pXO1, pXO2, bams22, bams23, bams30, bams51. The fluorescent primers were made using WellRED D2, D3 and D4 (Sigma-Aldrich, Saint-Quentin Fallavier, France). The six additional loci (vntr12 (labeled with D4), vntr16 (D2), vntr17 (D3), vntr19 (D2), vntr23 (D3), vntr35 (D3)) were multiplexed in a single PCR subsequently called E. The PCR products were diluted 1∶10 and 2 µl of the dilution were added to a mix containing 30 µl of Sample Loading Solution (SLS, Beckman Coulter, Fullerton, CA., USA) and 0.25 µl of size marker. The samples were separated by electrophoresis in CEQ Separation Gel LPA I on a CEQ 8000 automatic DNA Analysis System (Beckman Coulter, Fullerton, CA., USA) under the following conditions: denaturation at 90°C for 120 sec, injection at 2.0 kV for 30 sec, separation at 4.8 kV for 60 min (multiplexes A, C, E) or 6 kV for 70 min (multiplexes B and D). Allele sizes were estimated using the CEQ Fragment Analysis System software, by comparing the amplicon to the size marker “DNA Size standard kit-600” (multiplexes A, C, E; Beckman Coulter, Fullerton, CA., USA) or a custom-made “CST D1 100–1150 bp” (multiplexes B, D; Bioventures, Murfreesboro, TN, USA).

**Table 1:** List of 32 published *B. anthracis* VNTR markers.

Locus	Forward primers (5′-3′)	Reverse primers (5′-3′)	Comment
vrrA_12bp_314bp_4U^a^	CACAACTACCACCGATGGCACA	GCGCGTTTCGTTTGATTCATAC	
vrrB1_9bp_229bp_20U^a^	ATAGGTGGTTTTCCGCAAGTTATTC	GATGAGTTTGATAAAGAATAGCCTGTG	
vrrB2_9bp_153bp_13U^a^	CACAGGCTATTCTTTATCAAACTCATC	CCCAAGGTGAAGATTGTTGTTGA	
vrrC1_9bp_580bp_53U^a^	GAAGCAAGAAAGTGATGTAGTGGAC	CATTTCCTCAAGTGCTACAGGTTC	
vrrC2_18bp_532bp_17U^a^	CCAGAAGAAGTGGAACCTGTAGCAC	GTCTTTCCATTAATCGCGCTCTATC	
CG3_5bp_158bp_2U^a^	TGTCGTTTTACTTCTCTCTCCAATAC	AGTCATTGTTCTGTATAAAGGGCAT	
pXO1-aat_3bp_126bp_7U^a^	CAATTTATTAACGATCAGATTAAGTTCA	TCTAGAATTAGTTGCTTCATAATGGC	pXO1 Plasmid
pXO2-at_2bp_141bp_9U^a^	TCATCCTCTTTTAAGTCTTGGGT	GTGTGATGAACTCCGACGACA	pXO2 Plasmid
bams01_21bp_485bp_16U^b^	GTTGAGCATGAGAGGTACCTTGTCCTTTTT	AGTTCAAGCGCCAGAAGGTTATGAGTTATC	Alias vntr32^c^
bams03_15bp_549bp_26U^b^	GCAGCAACAGAAAACTTCTCTCCAATAACA	TCCTCCCTGAGAACTGCTATCACCTTTAAC	
bams05_39bp_307bp_5U^b^	GCAGGAAGAACAAAAGAAACTAGAAGAGCA	ATTATTAGCAGGGGCCTCTCCTGCATTACC	
bams07_18bp_1017bp_50U^b^ *	GAATATTCGTGCCACCTAACAAAACAGAAA	TGTCAGATCTAGTTGGCCCTACTTTTCCTC	bclE^e^
bams13_9bp_814bp_70U^b^	AATTGAGAAATTGCTGTACCAAACT	CTAGTGCATTTGACCCTAATCTTGT	bclA^e^
bams15_9bp_418bp_24U^b^	GTATTTCCCCCAGATACAGTAATCC	GTGTACATGTTGATTCATGCTGTTT	bclD^e^
bams21_45bp_676bp_10U^b^	TGTAGTGCCAGATTTGTCTTCTGTA	CAAATTTTGAGATGGGAGTTTTACT	
bams22_36bp_735bp_16U^b^	ATCAAAAATTCTTGGCAGACTGA	ACCGTTAATTCACGTTTAGCAGA	
bams23_42bp_651bp_11U^b^	CGGTCTGTCTCTATTATTCAGTGGT	CCTGTTGCTCCTAGTGATTTCTTAC	
bams24_42bp_595bp_11U^b^	CTTCTACTTCCGTACTTGAAATTGG	CGTCACGTACCATTTAATGTTGTTA	
bams25_15bp_391bp_13U^b^	CCGAATACGTAAGAAATAAATCCAC	TGAAAGATCTTGAAAAACAAGCATT	
bams28_24bp_493bp_14U^b^	CTCTGTTGTAACAAAATTTCCGTCT	TATTAAACCAGGCGTTACTTACAGC	
bams30_9bp_727bp_57U^b^	GCATAATCACCTACAACACCTGGTA	CAGAAAATATTGGACCTACCTTCC	bclB^e^
bams31_9bp_772bp_64U^b^	GCTGTATTTATCGAGCTTCAAAATCT	GGAGTACTGTTTGTTGAATGTTGTTT	bclC^e^
bams34_39bp_503bp_11U^d^	CAGCAAAATCAATCGAATCAAA	TGTGCTAAATCATCTTGCTTGG	
bams44_39bp_417bp_8U^d^	GCGAATTAATTGCTCCTCAAAT	GCACTTGAATATTTGGCGGTAT	
bams51_45bp_493bp_9U^d^	ATTTCCTGAAGCAGGTTGTGTT	TGCATCTAACAATGCAGAACAA	
bams53_12bp_236bp_8U^d^	GAGGTGTGTTAGGTGGGCTTAC	CATATTTTCACCTTAATTTTGGAAG	
vntr12_2bp_115bp_6U^c^	CGTACGAAGTAGAAGTCATTAA	GCATATAATTGCACCTCATCTAG	
vntr16_8bp_273bp_20U^c^	CTCTTGAAAATATAAAACGCA	GAATAATAAGGGTTCTCATGGTAT	pXO2 plasmid
vntr17_8bp_386bp_4U^c^	TAGGTAAACAAATTTTCGTAATC	GATCGTACAACAGCAATTATCAT	pXO2 plasmid
vntr19_3bp_96bp_4U^c^	GTGATGAAATCGGACAAGTTAGGAG	GAAATATTTTATTAAACATGCTTTCCATCC	
vntr23_12bp_197bp_4U^c^	TTTAGAAACGTTATCACGCTTA	GTAATACGTATGGTTCATTCCC	
vntr35_6bp_115bp_5U^c^	AAATAATATGTTCCTTTTGCTG	GTCCTGAAATAAATGCTGAAT	

a Keim et al. 2000 [6];

b Le Flèche et al. 2001 [10];

c Keim et al. 2004 [15];

d Lista et al. 2006 [11];

e Leski et al. 2009 [43].

*Locus not currently used due to some large amplicon sizes [10]. The numerical allele coding convention is as published by Keim et al., [6], Lista et al. [11] and Antwerpen et al. [26], see Data S1 for more details on the coding conventions and comparison with alternative coding conventions.

### VNTR allele coding convention

We have used the numeric coding convention for the 8 loci constituting MLVA8 proposed by Keim et al. [6] except for vrrC1. vrrC1 was initially considered as a 36 bp repeat unit, but it varies in a more complex way. Lista et al. proposed to consider vrrC1 as a 9 bp repeat unit VNTR, and assigned a 53 U allele code to the Ames ancestor genome accession number NC_007530.2. We have used the Lista et al. proposal [11] for the additional 17 loci constituting MLVA25 and Antwerpen et al. [26] for the additional 6 loci constituting MLVA31 (including MLVA15 by Van Ert et al. [8]; see Data S1 for more details on the coding conventions and comparison with alternative published coding conventions). Datasets using different conventions can be adjusted as far as the coding convention applied *in silico* to a sequenced genome is indicated. The convention as applied *in silico* to *B. anthracis* strain ‘Ames Ancestor’ accession numbers NC_007530.2 (chromosome), NC_007322.2 (pXO1 plasmid) and NC_007323.2 (pXO2 plasmid) is recalled in Table 1 and Data S1 and S2.

### Discriminatory power

The discriminatory power was calculated by using Simpson's diversity index (DI) [34]. A DI-value of 1 indicates that the typing method is able to discriminate between all isolates. A DI-value of 0 indicates that all isolates are identical. The discriminatory power of individual loci was calculated on a dataset comprising only one strain per MLVA31 genotype. The discriminatory power of the different MLVA panels was calculated on the full dataset.

### Data analysis

MLVA clustering was performed using the BioNumerics software package version 6.6 (Applied-Maths, Sint-Martens-Latem, Belgium). Data were analysed as a character dataset using the categorical distance coefficient. Clustering was achieved either with UPGMA (Unweighted Pair Group Method with Arithmetic mean) or Minimum Spanning Tree (MST). The priority rule for constructing MST was set so that the type that had the highest number of single-locus variants (SLVs) would be linked first. A cutoff value of 85% of similarity was applied to define clonal complexes (CC).

Congruence between different experiments was measured by BioNumerics using the categorical distance coefficient. Linkage disequilibrium was measured by using LIAN version 3.5 software (http://guanine.evolbio.mpg.de/) as described by Haubold & Hudson [35]. The linkage disequilibrium analysis for each MLVA panel was conducted on a dataset comprising one strain per MLVA genotype. The Monte-Carlo simulation was run with 10000 iterations.

The contribution of each marker to the global standardized index of association (I_A_^S^) was calculated as the difference between the MLVA31 I_A_^S^ and the MLVA30 I_A_^S^ without this marker.

Suggestions for optimized panels were determined using the Automated Selection of Typing Target Subsets (AuSeTTS) Analysis [36].

### Online database

MLVA31 data generated in this study can be accessed in the “*Bacillus anthracis*” database at MLVAbank http://mlva.u-psud.fr/. MLVAbank is a demo project first described by Le Flèche et al., 2002 [37] and the previous major modifications were described by Grissa et al., 2008 [29]. The 2008 MLVAbank version allowed the making by registered users of private or public MLVA databases (registration is free). Unregistered users can query public databases. For the present project, three major functionalities have been added. Firstly MLVAbank can now store any kind of genotyping data, including multiple locus sequence typing (MLST), SNPs, and Clustered Regularly Interspaced Short Palindromic Repeats (CRISPR) derived data [38]. Secondly, different databases can be declared as being part of a cooperative database, after a number of conventions have been agreed upon, regarding field names, genotyping markers names, allele coding conventions. Thirdly, an *in silico* typing tool has been included, which can deduce MLVA and SNP genotypes from whole genome sequence data. The application is coded using Javascript. The input is PCR primers information (for MLVA typing) or SNP positions. A tutorial is available via the MLVAbank web page, and an overview is presented as File S3.

MLVAbank provides minimum dendrogram tracing tools, based on the categorical distance coefficient and UPGMA method. In addition MEGA format distance matrix or Newick format tree text files can be exported for analysis with the MEGA software [39] and FigTree [http://tree.bio.ed.ac.uk] respectively.

## Results

### MLVA31 on 130 strains of B. anthracis

**Figure 1:**
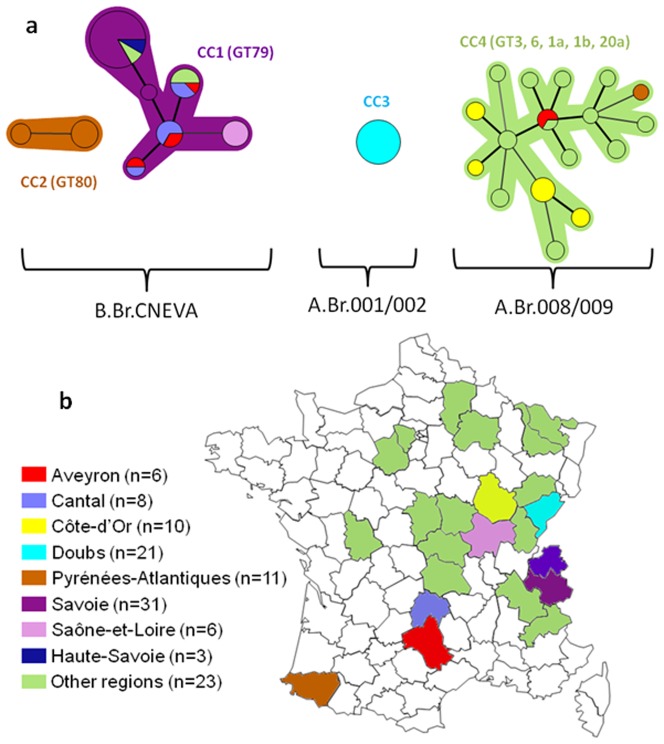
(a) Minimum spanning tree of MLVA31 data from 119 animal and environmental *B. anthracis* strains. Each circle represents a unique genotype. The diameter of each circle corresponds to the number of isolates with the same genotype. Genotypes connected by a shaded background differ by a maximum of 3 of the 31 VNTR markers and could be considered as a “clonal complex”. Thick connecting lines represent one locus differences; regular connecting lines represent two loci differences. The length of each branch is proportional to the number of differences. Each epidemiological situation is represented by a specific color as defined in part b.(**b**). Localization of the 119 animal and environmental *B. anthracis* strains.

**Figure 2:**
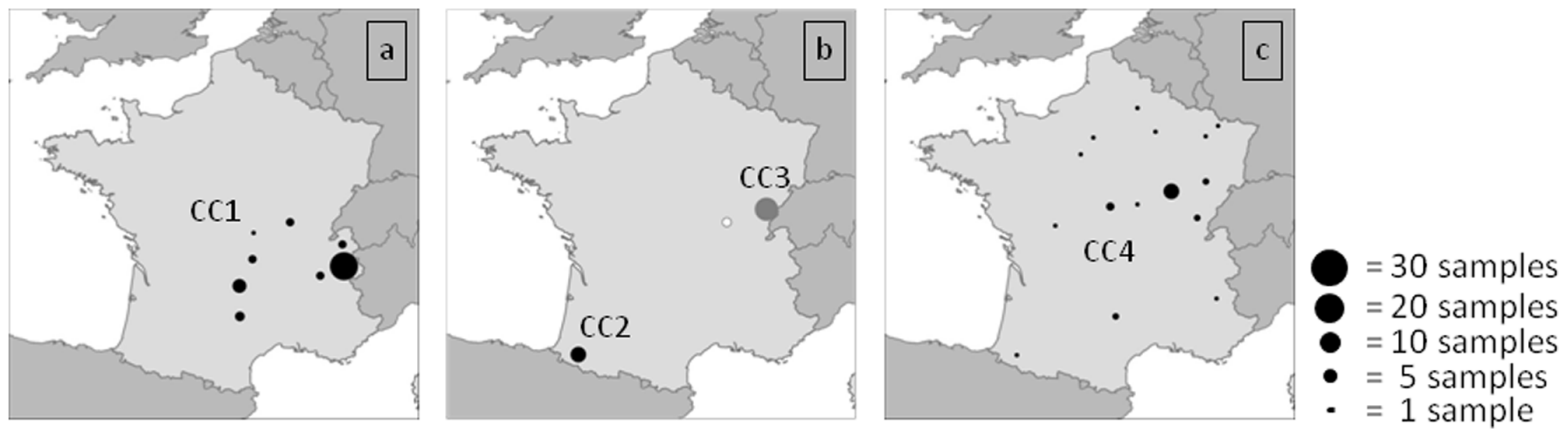
Geographical repartition of the strains for each clonal complex (CC). ^a^CC1 strains. ^b^CC2 (black circle), and CC3 (grey circle) strains. ^c^CC4 strains.

The discriminatory power of MLVA31 in this collection is 0.8874 (confidence interval 0.8491–0.9257) as compared to 0.7259 (0.6672–0.7846) for MLVA8, 0.8370 (0.8065–0.8675) for MLVA15 and 0.8831 (0.8436–0.9226) for MLVA25. MLVA31 resolves 35 genotypes (Data S2), fourteen contain more than one strain. The eleven external collection strains contribute nine genotypes whereas the 119 strains collected in France during the last three decades fall into 26 genotypes distributed in four clusters (minimum spanning tree, Figure 1a; the geographic origin of the strains is shown in Figure 1b). The geographical repartition of the different genotypes and clonal complexes (CCs) is presented on the map of France (Figure 2 and Data S4) which illustrates the congruence between geographic origin and genetic clustering.

The largest cluster is CC1 and corresponds to MLVA8 GT79 [6], [17]. Seventy-five percent of the strains belonging to CC1 (44 out of 59) originate from three *départements* (Savoie and Haute-Savoie in the Alpes; Cantal in Massif Central). Strains from nearby Saône-et-Loire (n = 6), Puy-de-Dôme (n = 3), Isère (n = 3), Aveyron (n = 4) and Allier (n = 1) also cluster within CC1. Six genotypes compose this major cluster. All strains but one from Pyrénées-Atlantiques (ten out of eleven) constitute CC2 which also corresponds to MLVA8 GT80. CC3 includes all strains from Doubs (n = 21). CC3 and the A.Br.001/002 subgroup appear as minor groups that are geographically restricted to the east of France. Field specimens clustered into CC3 were all isolated from a recent episode associated with the death of 39 animals in the Doubs *département*.

CC4 is the most diverse cluster and corresponds to MLVA8 GT3, 6, 1a, 1b and 20a [6], [17]. It accounts for 17 genotypes, comprising 27 strains including all ten strains from Côte-d'Or, two strains from Aveyron, one of the eleven strains from Pyrénées-Atlantiques and 15 strains from various regions. Four external collection strains appear to be related to CC4 (CIP A204, CIP A205, CIP A206 and 17JB) whereas the seven others are genetically distinct from the four CCs (data not shown). The geographic localization of strains of each CC is illustrated in Figure 2. Fifty-nine percent (71 of the 119 strains) occurred in four *départements* (Savoie, Pyrénées-Atlantiques, Doubs, Cantal). The CC1 is primarily found in south-eastern France (Figure 2a) while CC2 and CC3 are restricted to a single area (Figure 2b). Genotypes clustered within CC4 are observed throughout the country (Figure 2c), and are associated with sporadic outbreaks.

### MLVA31 and canonical SNP analysis comparison

Most strains (128 among 130 strains) belong to three canSNP subgroups, A.Br.001/002, A.Br.008/009, and B.Br.CNEVA. The last two are external collections strains belonging respectively to lineages A.Br.005/006 and A.Br.Vollum not naturally present in France [40]. Strains clustered within CC1 and CC2 are affiliated with the B.Br.CNEVA sub-lineage, while strains clustered within CC3 and CC4 are part of the A.Br.001/002 and A.Br.008/009 sub-groups, respectively (Figure 1a).

B.Br.CNEVA is the main sub-lineage found in France, with a majority of strains affiliated with this lineage. Bams01, vrrB1, bams15 and bams23 appeared as defining diagnostic markers for the sub-lineage present in France, with unique number of repeat copies (14, 19, 42 and 10, respectively). Three other VNTR loci (bams22, bams34 or bams51) highlighted regional clustering patterns, suggesting a successful establishment and spatial differentiation of B.Br.CNEVA strains in France. For instance, strains from the Pyrénées (CC2) were characterized by 9 repeat units alleles for marker bams34 and 8 repeat units alleles for marker bams51, whereas strains from the Alps (CC1) had 13 repeat units for marker bams22.

The most diverse CC4 represents the second group of strains and two-thirds of the genotypes. French strains affiliated with CC4 and the TransEurasian group (A.Br.008/009) originated from episodic outbreaks occurring throughout the country, with a particular spot in the Côtes-d'Or *département*. All CC4 genotypes share the same CG3 allele previously described for most strains belonging to the A.Br.008/009 and A.Br.WNA canSNP types [15]. The five-nucleotide sequence of CG3 is present in only one copy. The most discriminative VNTR loci within CC4 were bams30 (six different alleles), followed by bams15, and bams05 (four different alleles). Here again, the exosporium coding VNTRs appear to be among the most variable genetic elements within *B. anthracis*.

### Individual VNTR markers evaluation

**Figure 3:**
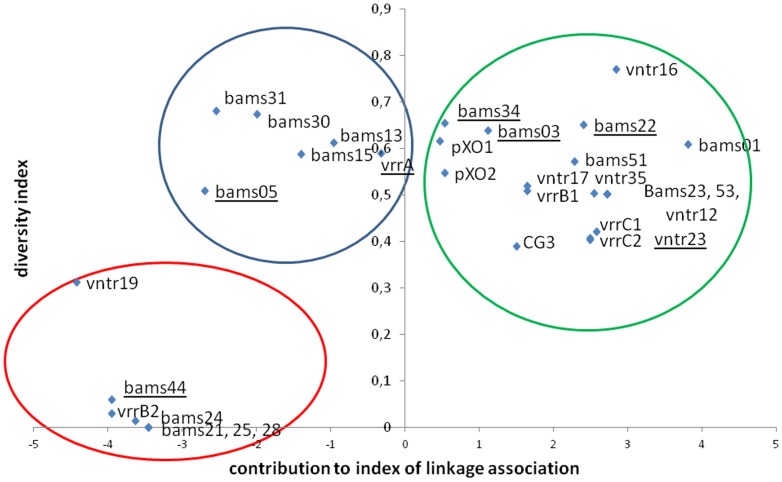
Graphic comparison of the discriminatory power and the contribution to the standardized index of association (I_A_^S^) for each VNTR marker.

The diversity index of each VNTR marker and contribution to the standardized index of association (I_A_^S^) was calculated on a dataset using one strain per MLVA31 genotype (Data S2 and Table 2). Three panels of markers can be distinguished (Figure 3). Panel A contains the 18 loci with positive I_A_^S^ contribution. Diversity indexes of these loci vary from 0.30 to 0.77. Panel B includes six markers (vrrA, bams05, bams13, bams15, bams30, bams31) with a similar diversity range but a negative contribution to I_A_^S^. Panel C is composed of seven markers showing negative contribution to I_A_^S^ and low diversity, comprising three monomorphic markers (bams21, bams25 and bams28) in addition to vrrB2, bams24, bams44 and vntr19.

**Table 2:** Discriminatory power and linkage disequilibrium analysis results for 5 sets of VNTR markers: the newly proposed MLVA7 and MLVA8, MLVA15, MLVA25, MLVA31.

	MLVA7	MLVA8	MLVA15	MLVA25	MLVA31	MLVA31*
Number of loci	7	8	15	25	31	31
Standardized index of association (*I_A_^S^*) French dataset	0.2323	0.1371	0.2300	0.3889	0.4166	ND
Diversity Index (*DI*) (genotypes)	0.8551 (19)	0.7259 (14)	0.8370 (17)	0.8831 (34)	0.8874 (35)	-
DI, Namibia dataset*	0.3111 (16)	0.7214 (23)	0.7214 (24)	0.7450 (38)	-	0.7450 (38)
vrrA	X	X	X	X	0.5895	0.1522
vrrB1		X	X	X	0.5091	0.2347
vrrB2		X	X	X	0.0306	0.0526
vrrC1		X	X	X	0.4078	0.1522
vrrC2		X	X	X	0.4036	0.1024
CG3		X	X	X	0.3893	0.1024
pXO1		X	X	X	0.6167	0.7112
pXO2		X	X	X	0.5478	0.8051
bams01			X	X	0.6093	0.4950
bams03	X			X	0.6382	0.2006
bams05	X			X	0.5095	0.4950
bams13				X	0.6130	0.6828
bams15				X	0.5883	0.5021
bams21				X	0.0000	0.1494
bams22	X			X	0.6503	0.3713
bams23				X	0.5020	0.5505
bams24				X	0.0154	0.0000
bams25				X	0.0000	0.0000
bams28				X	0.0000	0.0000
bams30				X	0.6739	0.4950
bams31				X	0.6809	0.6415
bams34	X			X	0.6549	0.4723
bams44	X			X	0.0601	0.5576
bams51				X	0.5723	0.0000
bams53				X	0.5020	0.0000
vntr12			X		0.5020	0.0526
vntr16			X		0.7710	0.1522
vntr17			X		0.5200	0.2802
vntr19			X		0.3131	0.1935
vntr23	X		X		0.4213	0.2404
vntr35			X		0.5038	0.1024

*Values as calculated from MLVA31 data published by [16].

### MLVA7 scheme

**Figure 4:**
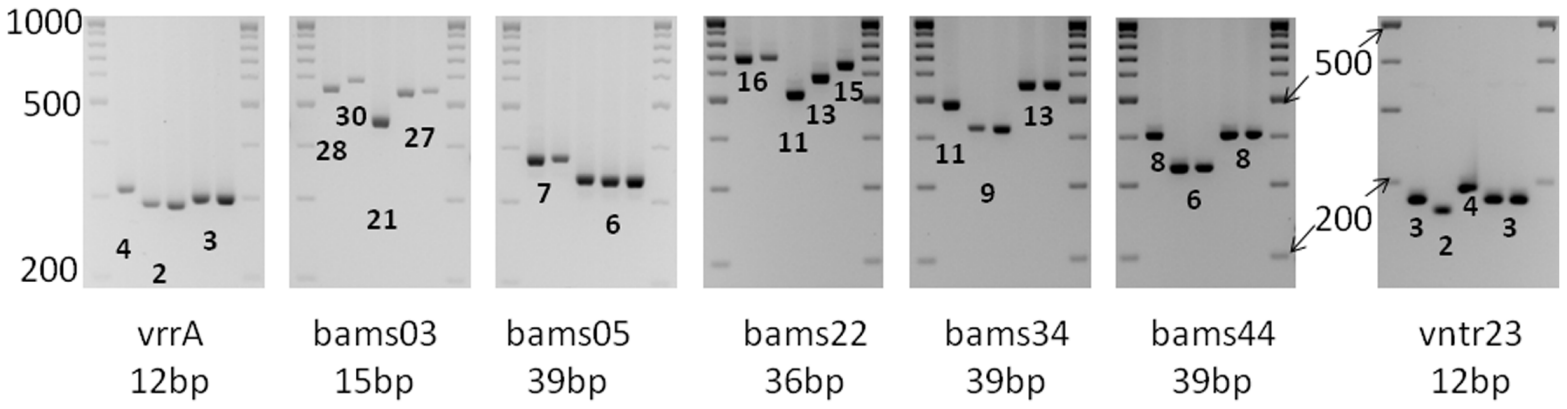
Electrophoresis gel of the MLVA7 panel on four strains. Well 1: Sterne strain, wells 2 to 4: French bovine strains. Migration on 3% standard agarose gel at 110 V during 4 hours. A 100 bp ladder was used running from 100 bp up to 1000 bp, the 500 bp and 1000 bp bands are more intense.

Marker evaluation allowed to select an optimised panel of seven VNTR markers among those most suitable for agarose gel typing, and the intermediate sizing resolution capillary electrophoresis such as Qiaxcel [41]. Sixteen loci have a repeat unit size (more than 10 bp) and observed allele size range compatible with agarose gel typing. The newly defined MLVA7 scheme includes the following agarose-friendly markers: vrrA, bams03, bams05, bams22, bams34, bams44, and vntr23 (Figure 4). None of these loci are located on the plasmids. Two are common to the MLVA15 assay. The MLVA7 panel resolves as many genotypes as the sixteen agarose-friendly loci, 14 genotypes in the 119 strains from France and 19 genotypes in the full collection of 130 strains (diversity index, 0.8584 confidence interval 0.8239–0.8929).

### Comparison of MLVA schemes (MLVA7, MLVA8, MLVA15, MLVA25 and MLVA31) on the “France” and “Namibia” MLVA datasets

**Figure 5:**
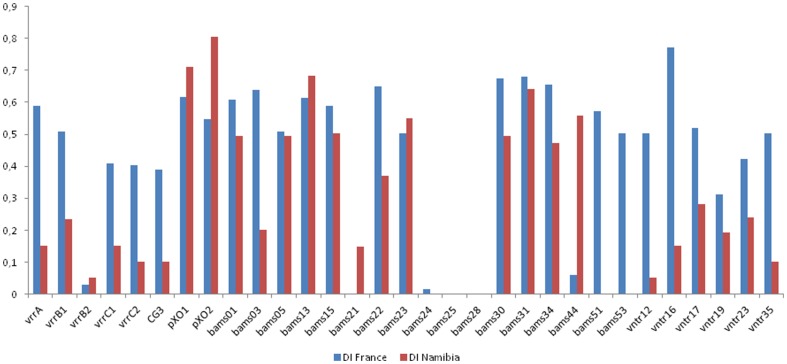
Comparison of the diversity index (DI) of individual loci in the “France” versus “Namibia” datasets.

Beyer et al. have published an extensive description of *B. anthracis* genetic diversity in Namibia as assayed by MLVA31 analysis [16]. The global diversity index of the assay calculated on the full dataset is recalled in Table 2, together with individual loci diversity calculated on a dataset comprising one strain per genotype. The situation in France and Namibia is similar with few canSNP lineages represented. Figure 5 illustrates the behavior of the different VNTR loci in both countries. Whereas some loci are similarly variable in both datasets (e.g. pXO1, pXO2, bams05, bams13 etc), others behave strikingly differently. Usually the diversity of this second group of VNTRs is higher in the French dataset, but this however simply reflects the presence of both cluster A and cluster B strains in the French dataset (Data S2).

The diversity index *DI* of each MLVA scheme calculated on both collections of strains and the standardized index of linkage association calculated keeping one strain per genotype are given in Table 2. The non-null standardized index of linkage association is in agreement with the clonal structure of the *B. anthracis* population.

**Figure 6:**
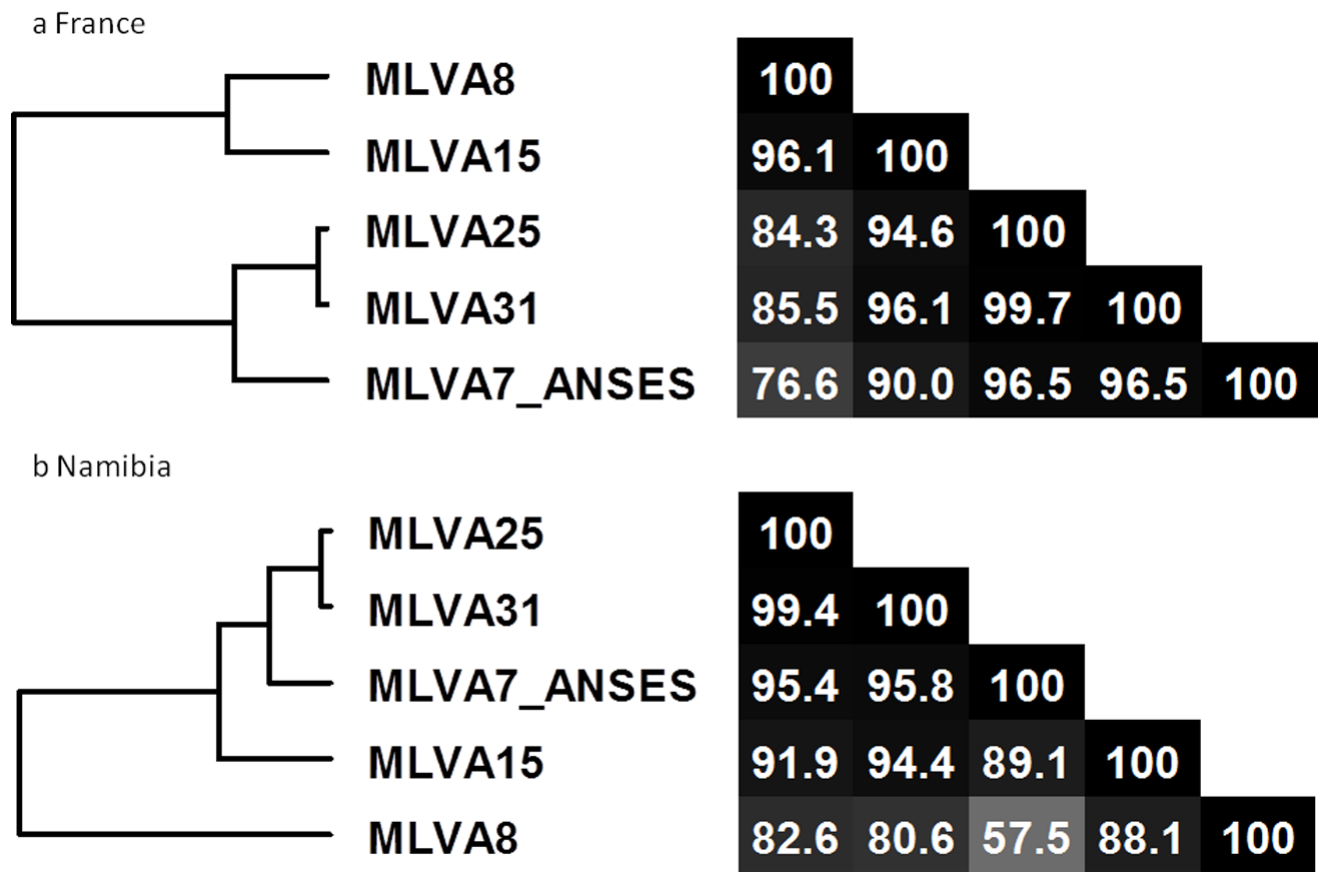
Congruence analysis of 5 sets of VNTR markers: MLVA7, MLVA8, MLVA15, MLVA25 and MLVA31. a congruence analysis in the “France” dataset (this study); b congruence analysis in the “Namibia” dataset [16].

Direct comparison of the different MLVA schemes showed a strong correlation between the clustering methods, as illustrated by the congruence analysis in Figure 6a for the “France” dataset. The congruence between MLVA31 and the MLVA25 scheme was 99.7% for the set of 130 strains typed. The congruence between MLVA31 and MLVA15 was slightly lower (96.1%) and much better than that determined with MLVA8 (85.5%). Interestingly MLVA7 demonstrated a discriminatory power (0.8584), I_A_^S^ value (0.2323) and congruence with MLVA31 (96.5%) higher than both MLVA8 and MLVA15.

The corresponding MLVA panels DI values deduced from the “Namibia” MLVA31 dataset [16] are indicated in Table 2. The global MLVA31 is significantly lower in the Namibia dataset as compared to the French dataset, but this might simply reflect a more extensive sampling in a geographically more focused area. The MLVA8 and MLVA15 DIs are almost as high, and this is largely due to the pXO1 and pXO2 loci. In comparison, MLVA7 has a much lower discriminatory power. Sixteen genotypes are resolved by MLVA7 (in comparison to 24 obtained with MLVA8 and MLVA15, and 38 with MLVA31). The maximum of 18 genotypes achievable with the 16 agarose-friendly loci would be reached by substituting bams21 to bams34, bams53 or vntr23. The congruence of MLVA7 with MLVA31 (95.8%) remains better as compared to MLVA8 or even MLVA15 (Figure 6b).

### Making of the MLVAbank *Bacillus anthracis* database

The *B. anthracis* database at http://mlva.u-psud.fr was updated taking advantage of the new version of the MLVAbank software, which allows the making of cooperative databases, the integration of SNP data, and the *in silico* analysis of whole genome sequence (Data S3). The current version includes a “*B_anthracis*_in_silico”, “*B_anthracis*_2012”, “*B_anthracis_*2013” and “*B_anthracis*_2014” components. *B_anthracis*_in_silico was deduced from the *in silico* MLVA and SNP typing of the seven fully sequenced strains released to date. It also includes *Bacillus cereus* and *Bacillus thuringiensis* genomes showing an average sequence similarity with *B. anthracis* above 97%. *B_anthracis*_2012 contains MLVA data recovered from articles on *B. anthracis* genotyping published up to year 2012, together with the associated SNP data. *B_anthracis_2013* contains data published in year 2013. *B_anthracis*_2014 presents the results available in Data S2.

Before inclusion, published data has been checked and normalized when necessary so that the same allele calling convention is used. For instance, Van Ert et al. [8] used a lettering convention for MLVA15, and the numbering convention proposed by Keim et al. 2000 [6] and Antwerpen et al. 2011 [26] was applied. Both Lista et al. 2006 [11] and Beyer et al. 2012 [16] used for some loci a convention that differed partly from previously published conventions, but the published data included *in silico* typing data from the Ames reference strain, allowing the recoding of the data set (see Data S1 for additional consideration on the MLVA31 published allele coding conventions).

## Discussion

Over the last decade, significant research efforts have been undertaken to develop appropriate genotyping methods for *B. anthracis* strain differentiation. The currently available methods take advantage of tandem repeat polymorphisms or point mutations. A typing strategy relying on a combination of genetic markers that are progressively less stable but have increasing resolving power (SNP, VNTRs, including SNRs) has been recommended [15]. In this system canSNPs typing is used to establish phylogenetic groups, which is followed by genotyping with MLVA. Once a representative set of strains will have been typed with both methods, MLVA data will be sufficient to robustly infer canSNP assignment. SNPs are evolutionary stable DNA signatures with low mutation rate (10^−10^ changes per nucleotide per generation) and two allelic states. In addition canSNPs typing can be applied to very low amounts of DNA, and/or degraded DNA, which can be essential in a forensic context [42]. VNTR loci are genomic regions with higher mutational rate (ranging from <10^−5^ to >10^−4^ insertion-deletion mutations per generation) and a higher number of possible allelic states (12 for the bams30 marker in this study) [15].

Different technologies can be used to assay these polymorphisms, including whole genome sequencing [6], [9], [10], [11], [12], [13], [14]. In the coming years, genotyping will increasingly be achieved via whole genome sequencing owing to the advent of massively parallel sequencing technologies. At present, the most affordable of these technologies are not yet able to confidently reconstruct tandem repeat arrays, at least the larger ones but this is expected to change soon. As most laboratories dealing with *B. anthracis* will only encounter a few strains per year, systematic whole genome sequencing will then turn out to be the most cost-efficient way to characterize these strains. One could even imagine that whole genome sequence information can then be used to decide if a strain is worth being kept in collection, or can be destroyed to avoid maintenance cost associated with this kind of highly dangerous pathogen.

However, there are a number of situations in which massively parallel sequencing will not remove the need for alternative, light-weight, fast and low cost genotyping tools. The first one is routine validation and control of strain identity for strain collection maintenance purposes. It may be necessary to have the capacity to genotype many colonies within the appropriate biosafety level laboratory, on a crude thermolysate. The second one is forensic microbiology, where traces of potentially degraded DNA must be analysed. In both contexts, targeted characterization of previously characterized polymorphic loci (VNTRs or SNPs) by PCR amplification might remain an essential tool.

In the present work, the diversity of French *B. anthracis* was analysed in detail with MLVA31. The 130 strains were resolved into 35 different genotypes and four clonal complexes. This is very similar to the situation reported previously in Namibia [16]. The discrimination power achieved by MLVA31 was clearly improved compared to the MLVA8 assay. Interestingly the four VNTRs coding for key components of the *B. anthracis* exosporium (bams13, bams15, bams30 and bams31 [43]) are among the most diverse loci in both MLVA31 datasets currently available (France and Namibia).

We tried next to optimize at least for the present collection of strains the number of VNTR loci required to genotype with useful resolution and accuracy the French diversity of strains and designed a shortened MLVA scheme. Based on the individual marker evaluation performed in this study, the more informative VNTR markers suitable for agarose gel-based analysis could be identified. This panel contains seven loci and can be typed either by monoplex PCR and agarose gel electrophoresis or by multiplexing in a single PCR followed by capillary gel electrophoresis as previously illustrated for *B. anthracis* and other species [11], [44], [45].

The MLVA7 assay is similarly efficient in both datasets, an excellent correlation between MLVA7 and MLVA31 results was observed. Despite its lower discriminatory power compared to MLVA31 the MLVA7 scheme appears as a good alternative for typing *B. anthracis* whenever capillary electrophoresis technology is not available or a quick genotype analysis must be performed. The proposed MLVA7 panel correctly identifies the main features of the population structure of the two distinct populations investigated here. For higher discriminatory power, two additional panels could be used, each one fitting in one multiplex PCR: the “exosporium loci” panel including bams13, bams15, bams30 and bams31 and the “plasmid loci” panel including pXO1, pXO2, vntr16, vntr17.

MLVA31 data generated in this study were deposited in the “*Bacillus anthracis*” database which can be accessed at http://mlva.u-psud.fr/. This is an open and collaborative database on *B. anthracis* that has been created to share all available MLVA (and SNP) typing data in a unique database for comparison testing and epidemiological studies. A VNTR and SNPs search tool is also provided to determine *in silico* the genotype of any strain based on its whole genome sequence.

In conclusion, the work undertaken in the present study updates the genetic landscape of *B. anthracis* diversity in France from previous publications [17], [40] and provides extended datasets about autochthonous strains that can be used for future epidemiological, epizootiological or preliminary forensic studies so that hypotheses can be made about strains origin. We expect that we have achieved now a correct coverage of the genetic diversity of *B. anthracis* naturally occurring in France and that the small assay which has been devised using this data can be confidently used as a routine, first line assay for typing new French isolates. This low-cost first line assay will be sufficient to recognize an abnormal exotic isolate. The second line assay in this strategy will be whole genome SNP analysis, as can be done with currently available massively parallel sequencing technologies.

Formal forensics investigations, as would be undertaken in the case of a bioterror event, will rely upon single nucleotide polymorphisms assayed via whole genome draft sequencing or large-scale SNP typing, rather than tandem repeats polymorphisms [46], [47]. However, we believe that routine genotyping, useful for disease surveillance, or daily strain collection monitoring and quality checking, will still benefit from a very low cost assay such as MLVA analysis, especially if it can be run on agarose gels after simple PCR amplifications as well as be deduced *in silico* from future generations of massively parallel sequence data.

The online database described in this study may provide the seed for the establishment of a larger collaborative aggregation of databases from other research groups working on genotyping of *B. anthracis* strains.

## Supporting Information

Data S1 MLVA31 coding convention.
(DOC)

Data S2 Table of the 35 genotypes defined by the 130 *Bacillus anthracis* strains and external collections strains typed with the MLVA31 panel.
(XLS)

Data S3 Overview of MLVAbank.
(PPT)

Data S4 UPGMA analysis of the 119 French *Bacillus anthracis* strains based upon MLVA31 data. The color code reflects geographic origin and is as in Figure 1. MLVA clonal complex, canSNP lineage and MLVA8 genotype as published in [17].
(PPT)
