# Supplementary material for: Genotyping of French Bacillus anthracis Strains Based on 31-Loci Multi Locus VNTR Analysis: Epidemiology, Marker Evaluation, and Update of the Internet Genotype Database
Source: PLoS One. 2014 Jun 5;9(6):e95131. doi: 10.1371/journal.pone.0095131 (PMC4046976; doi:10.1371/journal.pone.0095131)
Supplement: Data S1 — MLVA31 coding convention. (DOC) [file pone.0095131.s001.doc]

***Bacillus anthracis* MLVA typing convention**

**Background**

The currently known tandem repeats showing polymorphism in *Bacillus anthracis* have been initially described (including detailed information allowing locus identification) in four reports . Keim and colleagues have initially coded the alleles using the size and proposed a repeat unit correspondence for the MLVA8 loci . Subsequently a letter code was proposed for the MLVA15 loci (which includes MLVA8) . One of the MLVA15 loci, vntr32 corresponds to bams01 for which a coding convention was proposed by Lista et al 2006 . Repeat unit coding for the six additional loci VNTR12, 16, 17 19, 23, and 35 was proposed by Antwerpen et al 2011 .

Beyer et al. 2012 were the first to use all 31 loci, and have proposed an alternative coding convention for some loci, as can be seen in Table 1. Although conversion of allele size to repeat copy number might be expected to be a straightforward process when designing MLVA assays in general, there are ambiguities due to the fact that some tandem repeats are imperfect, and that the precise starting and ending points of a tandem repeat array cannot be determined in an absolute way. Also, when an non-integer number of repeat unit is present in a VNTR locus, the value has to be rounded to an integer value. Some authors will propose to round to the nearest integer, while others recommend to round up to the next highest integer value to limit the risk of eventually having to code some alleles as 0. These issues have been discussed in detail .

In order to propose an harmonized coding convention, and in the absence of an official body able to impose the use of such convention, we have followed the first published convention, except if it leads to inconsistencies. Published conventions are listed in Table 1.

**Comments about the loci constituting MLVA8:**

**vrrC1** was considered as a 36 bp repeat unit by Keim et al. 2000, and the Ames and Sterne allele size indicated is 583 bp instead of 580 bp. Also allele size does not vary as a multiple of 36 bp, for instance the allele size in NC_012581,*Bacillus anthracis* str. CDC 684 is 535 bp, i.e. a 45 bp difference. For this reason, vrrC1 has subsequently been considered as a 9 bp repeat unit locus, even if it is not the case *sensu stricto*. To our knowledge, the first coding convention proposed for this 9 bp repeat unit in the Ames ancestor genome was 53U .

**vrrC2**: Keim et al. observed three alleles at vrrC2, 532 bp, 604 bp and 607 bp. They interpret vrrC2 as a 18 bp repeat units locus, and code the 532 bp allele in Ames ancestor as 17. The second (604 bp) allele should then be coded 21. We propose to merge alleles 604 and 607 in a single allele bin called 21.

The **pXO1** allele size in the Ames strain was indicated as 123bp in Keim et al. 2000 , but the actual size in Ames ancestor NC_007530.2 is 126 bp (which corresponds to 7 U according to the coding convention by Keim et al. 2000).

**Comments about the bams and vntr loci coding convention**

The disagreements between Lista et al. 2006 , Antwerpen et al. 2011 on one end, and Beyer et al. 2012 on the other end are shown in Table 1. In most cases they are +-1 U or +-2 U disagreements which probably result from different definitions of the start and end position of the tandem repeats (which will depend for example of the parameters used when applying Tandem Repeats Finder ). We propose to stick to the "first published" rule as done previously, and will only comment two more striking disagreements, VNTR16 and Bams25.

**VNTR16** with an 8 bp repeat unit was coded 20U by Antwerpen et al and 8U by Beyer et al. Strictly speaking, Beyer et al. are right, the 273 bp allele in Ames ancestor does not contain 20 repeat units. However, van Ert et al. 2007 describe a 143 bp allele in a significant number of strains. This size, 130 bp shorter than the Ames ancestor allele, presumably resulting from a secondary deletion event rather than a tandem repeat variation, would need to be assigned a negative value. By using a 20U convention code, Antwerpen et al. make it possible to code the 143 bp allele as 4U (16U difference corresponds to 128 bp). This is a coding convention compromise.

**Bams25** was coded 13U by Lista et al. 2006 and 4U by Beyer et al. 2012. The reason for the 13U convention results from Tandem Repeat Finder analysis (with parameters 2, 3, 5).

Indices: 4915015--4915210 Score: 70

Period size: 15 Copynumber: 12.5 Consensus size: 15

4915005 GGTTTTTTAT

* ** *

4915015 CTTCTTTTTTAGGTG

1 CTTCTTCTTGCGCTG

* **

4915030 CTTCTTTTTTAGGCT-

1 CTTC-TTCTTGCGCTG

* **

4915045 CTTCCTTTTTAGGCT-

1 CTT-CTTCTTGCGCTG

* *

4915060 CTTCTTTTTGAGCTTG

1 CTTCTTCTTGCGC-TG

** * **

4915076 TCCACCGTTGTTTGCTGGCG

1 --CTTC-TT-CTTGC-GCTG

* *** * *

4915096 CTGCTGGCTGAGCAG

1 CTTCTTCTTGCGCTG

* * ** *

4915111 GTGCCGCTTGTGCTG

1 CTTCTTCTTGCGCTG

* *** *

4915126 GTTGAGCTTGCGCATC

1 CTTCTTCTTGCGC-TG

* ** *

4915142 TTTAGCAGCTTGCTCTG

1 CTT--CTTCTTGCGCTG

* * *

4915159 CTTGTTTTTGTGCTG

1 CTTCTTCTTGCGCTG

** * *

4915174 CAGCTTCTTGTGCAG

1 CTTCTTCTTGCGCTG

* *

4915189 CTTTTTCTTGCGCTT

1 CTTCTTCTTGCGCTG

*

4915204 CTTGTTC

1 CTTCTTC

4915211 AGCAATACGT

**Table 1 *In silico* coding convention for Ames ancestor accession numbers NC_007530.2 (chromosome), NC_007322.2 (pXO1 plasmid) and NC_007323.2 (pXO2 plasmid)**

| **Locus*** | **Coding convention Keim et al. 2000** | **Coding convention Lista et al. 2006** | **Coding convention Antwerpen et al. 2011** | **Coding convention Beyer et al. 2012**** | **Proposed 2014 rule** |
| --- | --- | --- | --- | --- | --- |
| vrrA_12bp_314bp_4U | 4 | 10 | 10 | 4 | 4 |
| vrrB1_9bp_229bp_20U | 20 | 16 | 16 | 17 | 20 |
| vrrB2_9bp_153bp_13U | presumably 13 | 6 | 6 | 13 | 13 |
| vrrC1_9bp_580bp_53U | Coded 36 bp and other pbs | 53 | 53 | 53 | 53 |
| vrrC2_18bp_532bp_17U | 17 | 17 | 17 | 15 | 17 |
| CG3_5bp_158bp_2U | 2 | 2 | 2 | 2 | 2 |
| pXO1-aat_3bp_126bp_7U | 7 and size issue Ames 123bp-126bp |  | 7 | 6 | 7 |
| pXO2-at_2bp_141bp_9U | 9 |  | 9 | 9 | 9 |
| bams01_21bp_485bp_16U |  | 16 | 16 | 16 | 16 |
| bams03_15bp_549bp_26U |  | 26 |  | 26 | 26 |
| bams05_39bp_307bp_5U |  | 5 |  | 5 | 5 |
| bams13_9bp_814bp_70U |  | 70 |  | 70 | 70 |
| bams15_9bp_418bp_24U |  | 24 |  | 25 | 24 |
| bams21_45bp_676bp_10U |  | 10 |  | 9 | 10 |
| bams22_36bp_735bp_16U |  | 16 |  | 13 | 16 |
| bams23_42bp_651bp_11U |  | 11 |  | 10 | 11 |
| bams24_42bp_595bp_11U |  | 11 |  | 9 | 11 |
| bams25_15bp_391bp_13U |  | 13 |  | 4 | 13 |
| bams28_24bp_493bp_14U |  | 14 |  | 14 | 14 |
| bams30_9bp_727bp_57U |  | 57 |  | 57 | 57 |
| bams31_9bp_772bp_64U |  | 64 |  | 64 | 64 |
| bams34_39bp_503bp_11U |  | 11 |  | 9 | 11 |
| bams44_39bp_417bp_8U |  | 8 |  | 8 | 8 |
| bams51_45bp_493bp_9U |  | 9 |  | 9 | 9 |
| bams53_12bp_236bp_8U |  | 8 |  | 8 | 8 |
| vntr12_2bp_115bp_6U |  |  | 6 | 6 | 6 |
| vntr16_8bp_273bp_20U |  |  | 20 | 8 | 20 |
| vntr17_8bp_386bp_4U |  |  | 4 | 4 | 4 |
| vntr19_3bp_96bp_4U |  |  | 4 | 4 | 4 |
| vntr23_12bp_197bp_4U |  |  | 4 | 4 | 4 |
| vntr32 see bams01 |  |  |  |  |  |
| vntr35_6bp_115bp_5U |  |  | 5 | 4 | 5 |

* changes or differences with respect to first published convention are underlined.

** according to Table S1 in Beyer et al. 2012. Note : bams15 25U in Ames ancestor is not in agreement with 44U assigned to 598 bp ( Table S2 in Beyer et al. 2012).

**References**

**1. Lista F, Faggioni G, Valjevac S, Ciammaruconi A, Vaissaire J, et al. (2006) Genotyping of *Bacillus anthracis* strains based on automated capillary 25-loci multiple locus variable-number tandem repeats analysis. BMC Microbiol 6: 33.**

**2. Le Flèche P, Hauck Y, Onteniente L, Prieur A, Denoeud F, et al. (2001) A tandem repeats database for bacterial genomes: application to the genotyping of *Yersinia pestis* and *Bacillus anthracis*. BMC Microbiol 1: 2.**

**3. Keim P, Price LB, Klevytska AM, Smith KL, Schupp JM, et al. (2000) Multiple-locus variable-number tandem repeat analysis reveals genetic relationships within *Bacillus anthracis*. J Bacteriol 182: 2928-2936.**

**4. Van Ert MN, Easterday WR, Huynh LY, Okinaka RT, Hugh-Jones ME, et al. (2007) Global genetic population structure of *Bacillus anthracis*. PLoS One 2: e461.**

**5. Antwerpen M, Ilin D, Georgieva E, Meyer H, Savov E, et al. (2011) MLVA and SNP analysis identified a unique genetic cluster in Bulgarian *Bacillus anthracis* strains. Eur J Clin Microbiol Infect Dis 30: 923-930.**

**6. Beyer W, Bellan S, Eberle G, Ganz HH, Getz WM, et al. (2012) Distribution and molecular evolution of *Bacillus anthracis* genotypes in Namibia. PLoS Negl Trop Dis 6: e1534.**

**7. Vergnaud G, Pourcel C (2009) Multiple locus variable number of tandem repeats analysis. Methods Mol Biol 551: 141-158.**

**8. Vergnaud G, Pourcel C (2006) Multiple locus VNTR (Variable Number of Tandem Repeat) Analysis (MLVA). In Molecular Identification, Systematics and Population Structure of Prokaryotes Edited by Stackebrandt E.: Springer-Verlag: 83-104.**

**9. Benson G (1999) Tandem repeats finder: a program to analyze DNA sequences. Nucleic Acids Res 27: 573-580.**
