# Supplementary material for: Genotyping of French Bacillus anthracis Strains Based on 31-Loci Multi Locus VNTR Analysis: Epidemiology, Marker Evaluation, and Update of the Internet Genotype Database
Source: PLoS One. 2014 Jun 5;9(6):e95131. doi: 10.1371/journal.pone.0095131 (PMC4046976; doi:10.1371/journal.pone.0095131)
Supplement: Data S3 — Overview of MLVAbank. (PPT) [file pone.0095131.s003.ppt]

## Slide 1
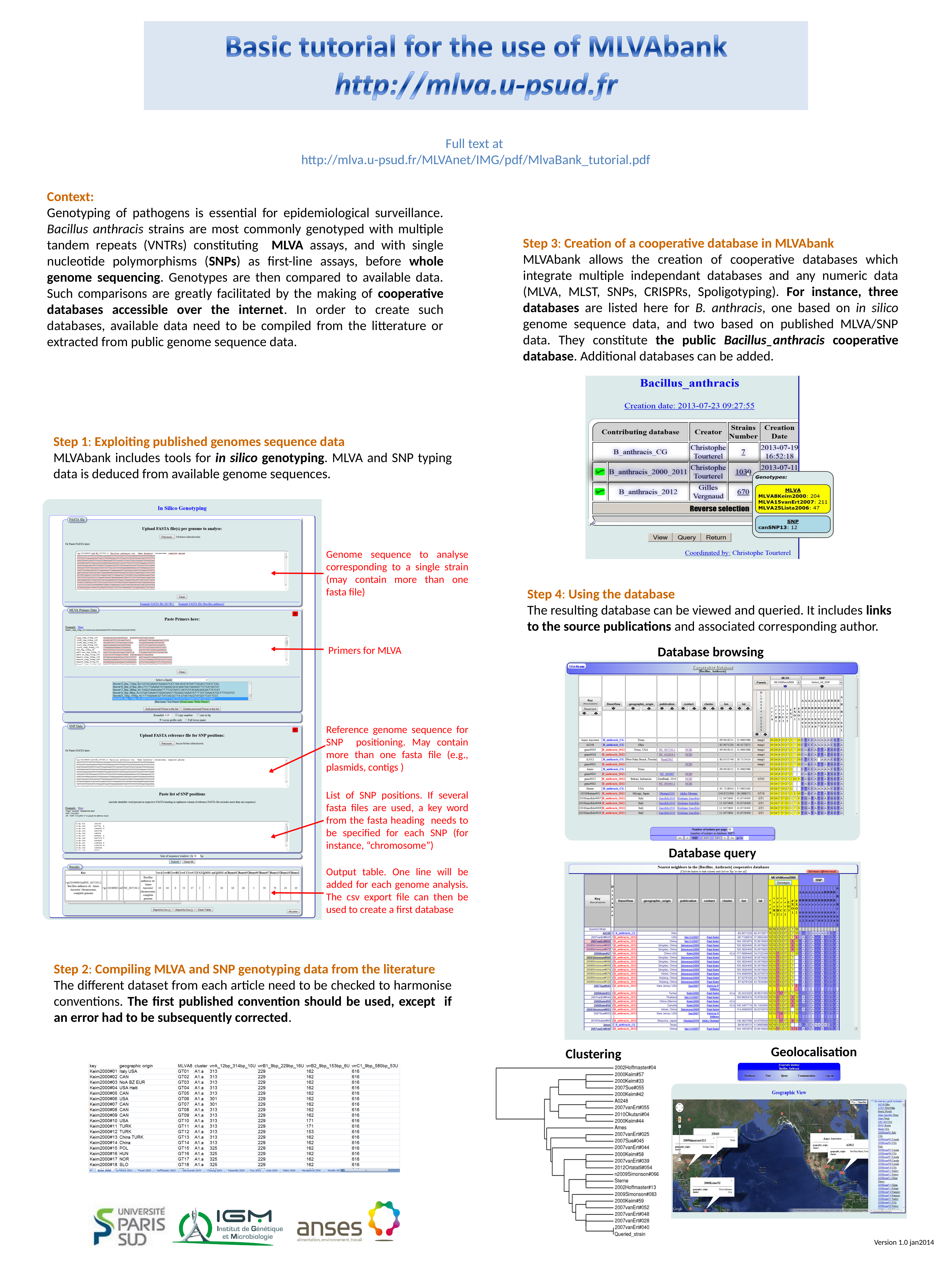

Full text at
http://mlva.u-psud.fr/MLVAnet/IMG/pdf/MlvaBank_tutorial.pdf
Context:
Genotyping of pathogens is essential for epidemiological surveillance. Bacillus anthracis strains are most commonly genotyped with multiple tandem repeats (VNTRs) constituting MLVA assays, and with single nucleotide polymorphisms (SNPs) as first-line assays, before whole genome sequencing. Genotypes are then compared to available data. Such comparisons are greatly facilitated by the making of cooperative databases accessible over the internet. In order to create such databases, available data need to be compiled from the litterature or extracted from public genome sequence data.
Step 3: Creation of a cooperative database in MLVAbank
MLVAbank allows the creation of cooperative databases which integrate multiple independant databases and any numeric data (MLVA, MLST, SNPs, CRISPRs, Spoligotyping). For instance, three databases are listed here for B. anthracis, one based on in silico genome sequence data, and two based on published MLVA/SNP data. They constitute the public Bacillus_anthracis cooperative database. Additional databases can be added.
Step 1: Exploiting published genomes sequence data
MLVAbank includes tools for in silico genotyping. MLVA and SNP typing data is deduced from available genome sequences.
Genome sequence to analyse corresponding to a single strain (may contain more than one fasta file)
Step 4: Using the database
The resulting database can be viewed and queried. It includes links to the source publications and associated corresponding author.
Database browsing
Primers for MLVA
Reference genome sequence for SNP positioning. May contain more than one fasta file (e.g., plasmids, contigs )
List of SNP positions. If several fasta files are used, a key word from the fasta heading needs to be specified for each SNP (for instance, “chromosome”)
Database query
Output table. One line will be added for each genome analysis. The csv export file can then be used to create a first database
Step 2: Compiling MLVA and SNP genotyping data from the literature
The different dataset from each article need to be checked to harmonise conventions. The first published convention should be used, except if an error had to be subsequently corrected.
Geolocalisation
Clustering
Version 1.0 jan2014
