# Supplementary material for: Genotyping of French Bacillus anthracis Strains Based on 31-Loci Multi Locus VNTR Analysis: Epidemiology, Marker Evaluation, and Update of the Internet Genotype Database
Source: PLoS One. 2014 Jun 5;9(6):e95131. doi: 10.1371/journal.pone.0095131 (PMC4046976; doi:10.1371/journal.pone.0095131)
Supplement: Data S4 — UPGMA analysis of the 119 French Bacillus anthracis strains based upon MLVA31 data. The color code reflects geographic origin and is as in Figure 1. MLVA clonal complex, canSNP lineage and MLVA8 genotype as published in [17]. (PPT) [file pone.0095131.s004.ppt]

## Slide 1
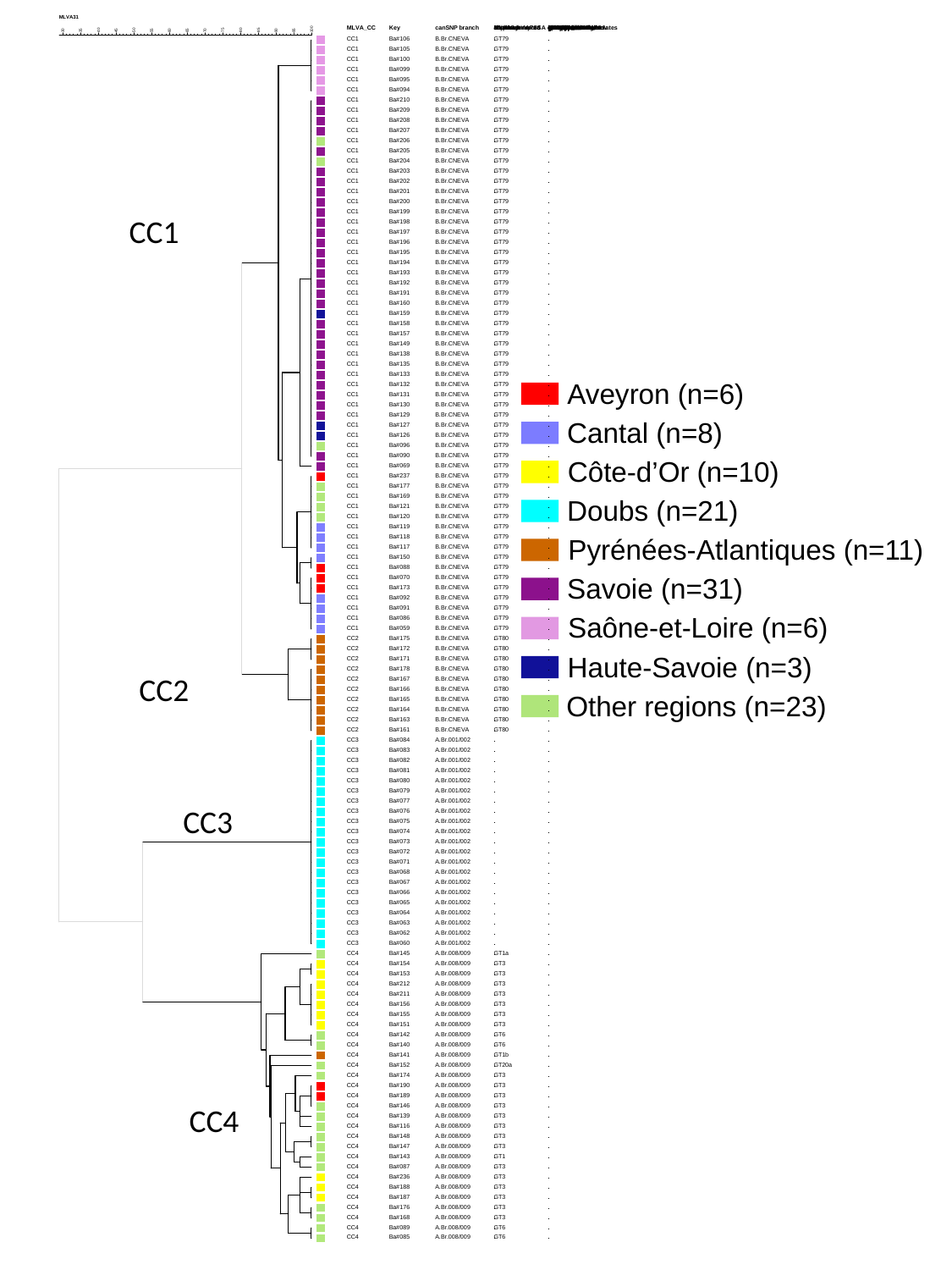

CC1
Aveyron (n=6)
Cantal (n=8)
Côte-d’Or (n=10)
Doubs (n=21)
Pyrénées-Atlantiques (n=11)
Savoie (n=31)
Saône-et-Loire (n=6)
Haute-Savoie (n=3)
Other regions (n=23)
CC2
CC3
CC4
